# Partial convergence of the human vaginal and rectal maternal microbiota in late gestation and early post-partum

**DOI:** 10.1038/s41522-023-00404-5

**Published:** 2023-06-13

**Authors:** Hakdong Shin, Keith A. Martinez, Nora Henderson, Melanie Jay, William Schweizer, Debby Bogaert, Gwoncheol Park, Nicholas A. Bokulich, Martin J. Blaser, Maria Gloria Dominguez-Bello

**Affiliations:** 1 Department of Food Science & Biotechnology, and Carbohydrate Bioproduct Research Center, College of Life Science, Sejong University, Seoul, South Korea; 2 Department of Medicine, New York University Langone Medical Center, New York, NY USA; 3 Department of Biochemistry and Microbiology, Rutgers University, New Brunswick, NJ USA; 4 Department of Population Health, New York University Langone Medical Center, New York, NY USA; 5 Department of Obstetrics and Gynecology, New York University Langone Medical Center, New York, NY USA; 6 MRC Centre for Inflammation Research, University of Edinburgh, Edinburgh, Scotland; 7 Laboratory of Food Systems Biotechnology, Institute of Food, Nutrition and Health, ETH Zürich, Zürich, Switzerland; 8 Center for Advanced Biotechnology and Medicine, Rutgers University, New Brunswick, NJ USA; 9 Department of Anthropology, Rutgers University, New Brunswick, NJ USA

**Keywords:** Microbiome, Clinical microbiology

## Abstract

The human vaginal and fecal microbiota change during pregnancy. Because of the proximity of these perineal sites and the evolutionarily conserved maternal-to-neonatal transmission of the microbiota, we hypothesized that the microbiota of these two sites (rectal and vaginal) converge during the last gestational trimester as part of the preparation for parturition. To test this hypothesis, we analyzed 16S *rRNA* sequences from vaginal introitus and rectal samples in 41 women at gestational ages 6 and 8 months, and at 2 months post-partum. The results show that the human vaginal and rectal bacterial microbiota converged during the last gestational trimester and into the 2nd month after birth, with a significant decrease in *Lactobacillus* species in both sites, as alpha diversity progressively increased in the vagina and decreased in the rectum. The microbiota convergence of the maternal vaginal-anal sites perinatally might hold significance for the inter-generational transmission of the maternal microbiota.

## Introduction

The maternal vaginal microbiome is a primordial source of microbes for the developing newborn^1–3^, carrying microbiotas from feces and other body sites^4^, providing seeding for multiple body sites in the baby, and protection against pathogens after birth^5^. For the mother, it remains important for health during and after birth^6^. Women undergo important physiological changes during gestation that also affect their microbiomes^7,8^. Vaginal *Lactobacillus* increase relative abundances during pregnancy and this is observed across American^9^, European^10^, African^11^, and Asian^12^ populations. The fecal microbiota also changes with pregnancy, with reduced richness, and increased abundances of Actinobacteria (including Bifidobacteriales) and Proteobacteria^7^. Third trimester fecal microbiota induce increased pro-inflammatory responses in germ-free mice compared with those from the first trimester^7^. The vaginal microbiota may have increased diversity post-partum, driven in part by increases in species of anaerobes (*Peptoniphilus*, *Prevotella*, and *Anaerococcus*), and a reduction in *Lactobacillus* species^9^.

Since in addition to the vagina, babies are exposed at birth to the mother’s perineum, which is a potential connection between the microbiomes of the intestine and the vagina, both of which undergo gestational changes. We hypothesized that the rectal and vaginal microbiotas converge during the last gestational trimester. This would be consistent with the conserved transformation of the maternal microbiota for transmission to the next generation. In this work, we determined the ante- and post-partum vaginal and rectal bacterial community structure in 21 mothers who delivered vaginally and in 20 mothers who delivered by Cesarean section (C-section), whose samples were obtained as part of an earlier study^13,14^.

## Results

### Comparison of vaginal and rectal communities during pregnancy and postpartum

**Fig. 1:**
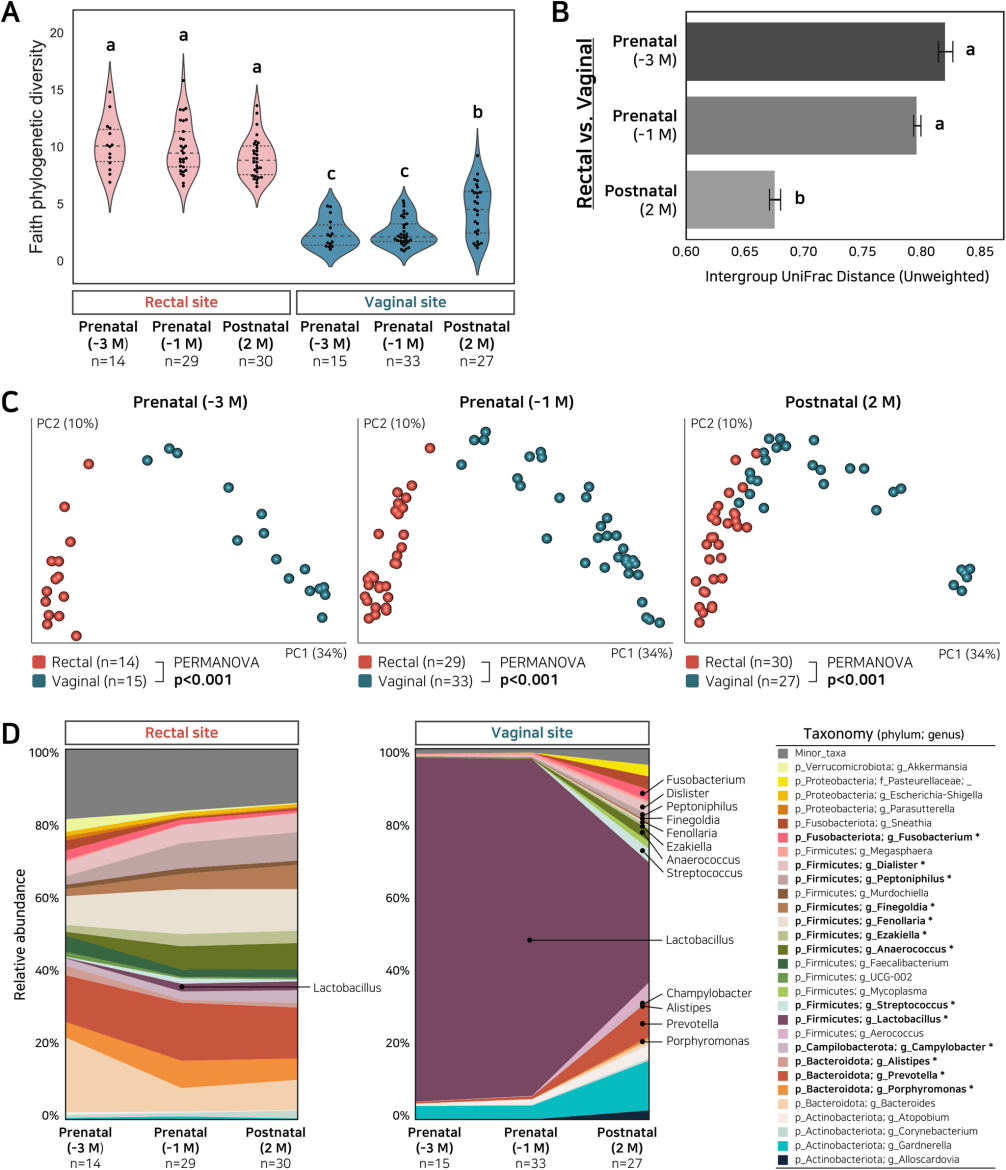
Diversity in maternal pre- and post-partum rectal and vaginal microbiomes in analyses of 16 S *rRNA* sequences. **A** Phylogenetic diversity in each site at three time-points. Labeled means without a common letter differ significantly, *p*-value < 0.05. Center line, median; quartile lines, upper and lower quartiles; violin plot, 1.5x interquartile range. **B** UniFrac distances between rectal and vaginal sites at 3 perinatal time-points. Error bars represent mean ± SEM. **C** PCoA of unweighted UniFrac distances between rectal and vaginal microbiomes at each time-point (PERMANOVA *p*-value < 0.001). **D** Taxa plots of maternal rectal (left panel) and vaginal (right panel) microbiomes at three peripartum time-points; *indicates LDA score >3.0 between –1M and +2 M.

We compared vaginal and rectal communities early (month 6) and late (month 8) in the third gestational trimester, and at 2 months post-partum, in 41 mothers (Supplementary Table 1 and Supplementary Table 2). A total of ~3 million V4 16S *rRNA* gene sequences with an average of ~20,000 sequences per sample were obtained (Supplementary Table 3). Bacterial alpha diversity in the rectum gradually declined over the last gestational trimester and continued to decrease into the second month postpartum. In contrast, there was a postpartum increase in phylogenetic diversity of vaginal microbiota (Fig. 1A), with increasing tendencies in either richness or evenness (Supplementary Fig. 1).

**Fig. 2:**
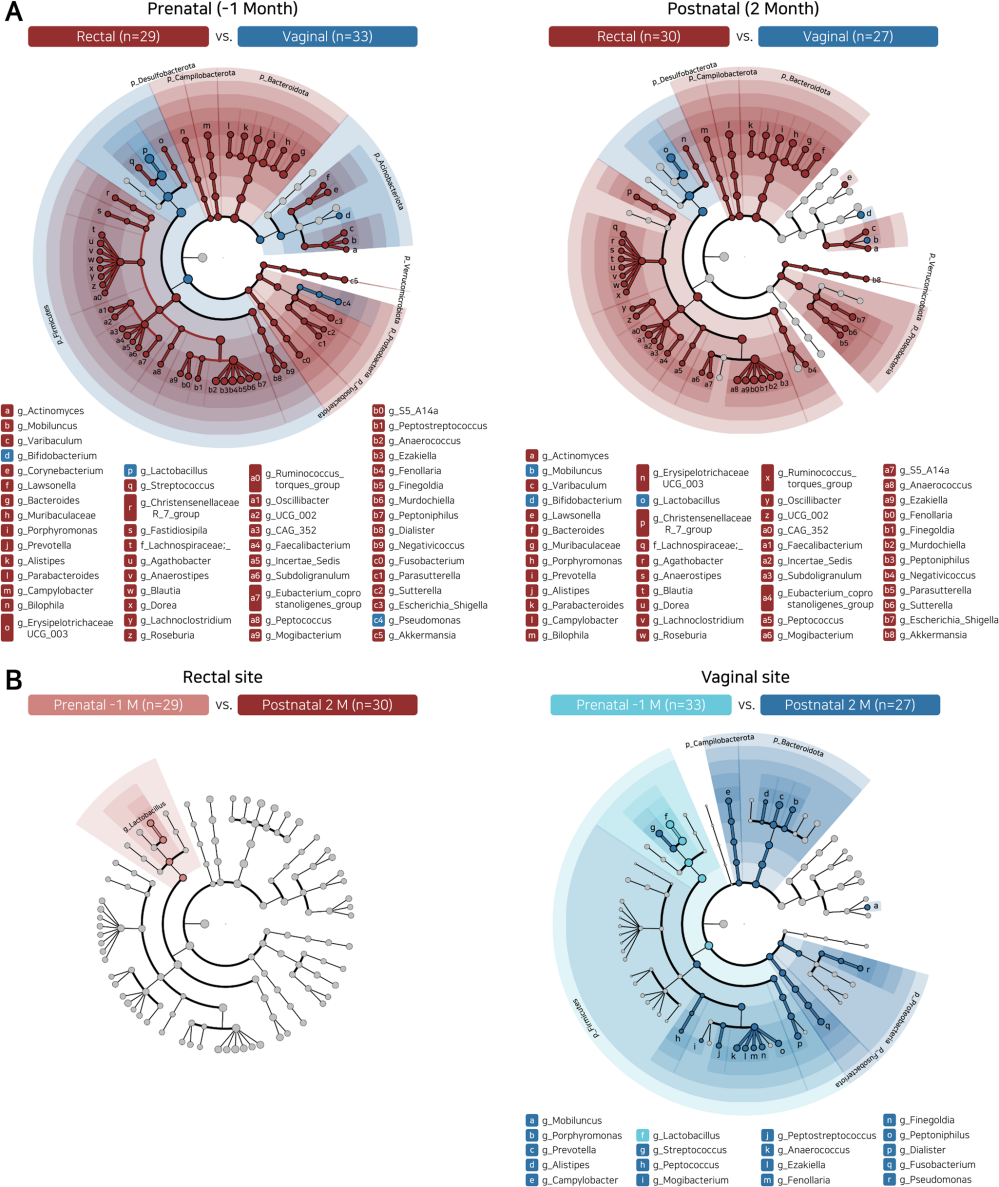
Differences in the bacterial taxa of maternal pre- and post-partum rectal and vaginal sites, based on 16 S rRNA high throughput sequencing. **A** LEfSe analysis of rectal versus vaginal sites late in gestation (left panel) or post-partum (right panel). **B** LEfSe analysis of prenatal versus post-partum bacterial taxa in rectal (left panel) or vaginal (right panel) sites.

More bacterial taxa differed significantly in relative abundance between vagina and rectum during the prenatal period (52 taxa) than in the postpartum period (45 taxa; Fig. 2A and Supplementary Fig. 2), consistent with convergence of community structure. Similarly, community distances between the rectal and vaginal microbiotas became progressively reduced from the last gestational trimester to post-partum month 2 (Figs. 1B, C). Paired bootstrapping analysis indicated that postpartum convergence was more pronounced in the vagina than in the rectum (Figs. 1D, 2, and Supplementary Fig. 3).

Temporal changes from prenatal to post-natal (Fig. 2) included reductions of *Lactobacillus* species relative abundances in both rectal and vaginal sites (Fig. 1D and Supplementary Fig. 4), and increases in Bacteroidetes including *Prevotella*, and in Firmicutes including *Streptococcus*, *Anaerococcus, Peptoniphilus*, and *Dialister* in the vaginal site (Fig. 2 and Supplementary Fig. 3). The predicted species for *Lactobacillus* features (DADA2-based) identified in this dataset were compared between vaginal and rectal microbiota. *Lactobacillus* features related to *L. iners, L. crispatus*, and *L. acidophilus* showed major components in shared microbial composition communities between the rectal and vaginal microbiotas. These temporal changes were more pronounced in vagina than in rectum, as evidenced by showing less error rates in supervised classification of vaginal samples by time-points, in relation to rectal samples (Supplementary Fig. 5 and Supplementary Fig. 6). The pre- or peripartum antibiotics exposure showed an equivalent tendency on vaginal/rectal microbial convergence (Supplementary Fig. 7).

## Discussion

Mammals evolved separate canals for reproduction, urination, and defecation but the rectal and vaginal orifices are typically proximal, while the urethra is not^15^. Because of their anatomical proximity and their perineal connectivity, we asked whether the two sites share bacteria during pregnancy. Our results show a clear convergence of the rectal and vaginal microbiota in the third trimester that extends post-partum. These results are consistent with independent reports of gestational changes in the vaginal^10–12,16^ and gut^7,9^ microbiota that were assessed separately, in different women. Although the vaginal microbiome varies with ethnicity, the gestational reduction in diversity abrogates these differences^17,18^. In the current study, we compared vaginal and rectal gestational changes longitudinally, in the same mothers, with consistent and robust changes, regardless of delivery mode. Microbial convergence found in this study reflects the increase in shared microbial composition communities, leading to higher community similarity, as evidenced by the reduced community distances between the rectal and vaginal microbiotas. Indeed, the convergence continued after birth, to post-partum month 2 (Fig. 1B–D) as shown by the results. Since rectal samples did not change in diversity, this convergence is not an artifact due to simply shallower sampling. The vaginal ecosystem converged towards the rectal one. We speculate that these higher similarities in vaginal/rectal microbial composition communities could lead to selection in favor of (i) a *Lactobacillus*-enriched vaginal microbiota that maximally protects the gravid uterus from invasive pathogens^19^, and (ii) mechanisms that will expose the neonate to the widest diversity of intestinal microbes to be transferred from the mother^2^. These biological roles of vaginal/rectal microbial convergence should be examined in further research. Limitations of this study included a small number of samples and a lack of control for the use of antibiotics during labor. Further studies should follow the maternal microbiota beyond 2 months post-partum and when it normalizes to the non-pregnant typical state.

Prior studies have shown that there is a large increase in beta-diversity in the maternal fecal microbiota in the third trimester, as the fecal microbial populations become more host-specific^7^. The post-labor vaginal microbiota differs from the baseline non-pregnant state, and the gestational changes have been shown to persist for up to 1 year after birth^9^.

Studies of mother-to-infant strain transmission show that the early infant gut microbiome contains maternal fecal bacteria^20^, further supporting our findings of vaginal/rectal microbiota convergence. Indeed, proof of concept of gut microbiota restoration in infants born by C-section have been shown using both vaginal^21^ and fecal^22^ maternal sources. In any case, elective C-sections also have the confounding effect of antibiotics for the procedure, and will impair the post-partum observations. Although different body sites of the baby during birth are exposed to the same maternal microbiome, baby site selection effect is observable at day 2 after birth, in the gut, skin and oral microbiota^21^, as the developmental succession continues after the infant’s birth^14^. Gestational changes in the microbiota might also be relevant to pregnancy outcomes^9^, since the fetus receives products of the metabolism of the maternal microbiota^23,24^, although the direction of causality is not well-understood.

Although the pre- and post-partum changes in the maternal microbial communities are clear, the significance of these for infant health remains unknown. We used DADA2-based methods to predict *Lactobacillus* species related to shared microbial composition communities between the rectal and vaginal microbiotas, and found that the major species were *L. iners, L. acidophilus*, and *L. crispatus*. *L. iners* has been associated with vaginosis^25^, and *L. crispatus* and *L. acidophilus* are associated with the high *Lactobacillus* dominance vaginal profile in healthy women^26^. If these changes optimize the exposure of infants to the beneficial maternal microbiota, they are of adaptive value. More research is needed to understand both the dynamics and functional significance of the maternal microbiota during the critical peripartum period of maternal recovery and child development and across wider racial demographics.

## Methods

### Sample and sequence information

In this study, we prospectively collected samples from the vaginal introitus and rectum of 41 women during pregnancy and at 2 months postpartum, as reported^13^. Vaginal -introitus- swabs were collected by the obstetrician and/or through self-collection by the mothers using sterile cotton-tipped swabs, a method shown to be reliable to quantify BV-associated bacteria^27^ and to identify vaginal bacterial structures^28^. For this analysis, we used rectal (*n* = 73) or vaginal (*n* = 75) samples from pregnant mothers in the early (weeks 23-35) or late (weeks 36-delivery) third gestational trimester and 2 months after delivery (Supplementary Table 1 and Supplementary Fig. 7). DNA had been extracted and the 16 S *rRNA* V4 gene region had been sequenced using the Illumina MiSeq platform, as reported^13^ (European Nucleotide Archive: PRJEB14529). The samples analyzed in this study consist of a subset of samples collected with informed consent according to a New York University Institutional Review Board-approved study that took place in New York City from 2011 to 2014. Participation was voluntary and included written informed consent.

### Data analysis

The 16S *rRNA* gene sequencing dataset, bacterial taxonomic compositions, and analyses of alpha-diversity (phylogenetic diversity^29^, observed ASVs, and ASV evenness) and beta-diversity (Unweighted/Weighted UniFrac distance^30^) were performed using QIIME 2 version 2020.6^31^ and its associated plugins. The q2-demux plugin was used for the demultiplexing and quality filtering of raw sequencing reads. Qualified reads were trimmed and denoised with DADA2^32^. All amplicon sequence variants (ASVs) were aligned using MAFFT^33^ and used to generate a rooted phylogenetic tree with FastTree 2^34^. The q2-feature-classifier plugin^35^ was used to trim the 99% SILVA 16S rRNA gene database^36^ to the 515F-806R (V4) region, train a naïve Bayes taxonomy classifier on these sequences, and use it to taxonomically classify each ASV. For comparisons of bacterial diversity, all communities were rarefied to 4347 reads (lowest number of reads) per sample, to include all samples the dataset. To determine significant differences in diversity, the Kruskal–Wallis test was used as a non-parametric test. Linear discriminant analysis (LDA) effect size (LEfSe) was used to detect statistically significant differences in the representation of bacterial taxa in comparisons (LDA score >3.0)^37^. Also, ALDEx2 tools^38^, which uses clr-transformed data generated from 128 Monte Carlo instances, used to confirm the LEfSe results. The Welch’s *t*-test was used followed by Benjamini–Hochberg false discovery rate (FDR) correction, and effect size was calculated. To evaluate community separation in beta diversity, PERMANOVA (with 999 random permutations)^30^ was used. Supervised classification of vaginal and rectal samples by time-points based on the ASVs table collapsed to genus level was performed using a q2-sample-classifier^39^ based on Random Forest classifier^40^ and nested stratified 5-fold cross-validation.

### Reporting summary

Further information on research design is available in the Nature Research Reporting Summary linked to this article.

## Supplementary information

Supplementary Information

Reporting Summary

## Data Availability

The sequence data have been deposited in the European Nucleotide Archive under accession number ERP016173.
