# Supplementary material for: Partial convergence of the human vaginal and rectal maternal microbiota in late gestation and early post-partum
Source: NPJ Biofilms Microbiomes. 2023 Jun 13;9:37. doi: 10.1038/s41522-023-00404-5 (PMC10264455; doi:10.1038/s41522-023-00404-5)
Supplement: Supplementary file 1 — Supplementary Information [file 41522_2023_404_MOESM1_ESM.pdf]

Supplementary Information

Supplementary Table 1. Distribution of 148 maternal samples in relation to time of parturition (3-, 1-month before delivery and 2-month after delivery).

Sample collection	Rectal (n samples)	Vaginal (n samples)
-3 Month	14	15
-1 Month	29	33
2 Month	30	27
Total	73	75

Supplementary Table 2. Baseline characteristics of the 41 mothers in the study

Characteristics	Mothers in study (n=41)<br>Mean ± SD or number (%)
**Race**	
Black or African American	2 (4.9)
White	27 (65.9)
Asian/Pacific Islander	7 (17.1)
Hispanic	4 (9.8)
Other	1 (2.4)
**Delivery mode**	
Vaginal delivery	21 (51.2)
C-section	20 (48.8)
**Pre-natal antimicrobial use**	**5 (12.2)**
**Peri-natal antimicrobial use (excluding C-section prophylaxis)**	**12 (29.3)**

Supplementary Table 3. Summary of analyzed sequence information.

Sample type	Rectal swabs			Vaginal swabs			Total
**Sampling time point**	**Prenatal (-3 Month)**	**Prenatal (-1 Month)**	**Postnatal (2 Month)**	**Prenatal (-3 Month)**	**Prenatal (-1 Month)**	**Postnatal (2 Month)**	
Number of samples	14	29	30	15	33	27	148
**Number of sequences**	**242,772**	**529,850**	**473,405**	**212,686**	**429,380**	**372,096**	**2,260,189**
Average sequence ± SD	17,341 ± 9,177	18,271 ± 8,202	15,780 ± 8,263	14,179 ± 4,134	13,012 ± 4,713	13,781 ± 4,635	15,272 ± 7,034
**Number of features**	**720**	**993**	**861**	**130**	**206**	**340**	**1,675**
Average features ± SD	118 ± 39	113 ± 40	95 ± 28	14 ± 8	15 ± 10	29 ± 21	63 ± 52

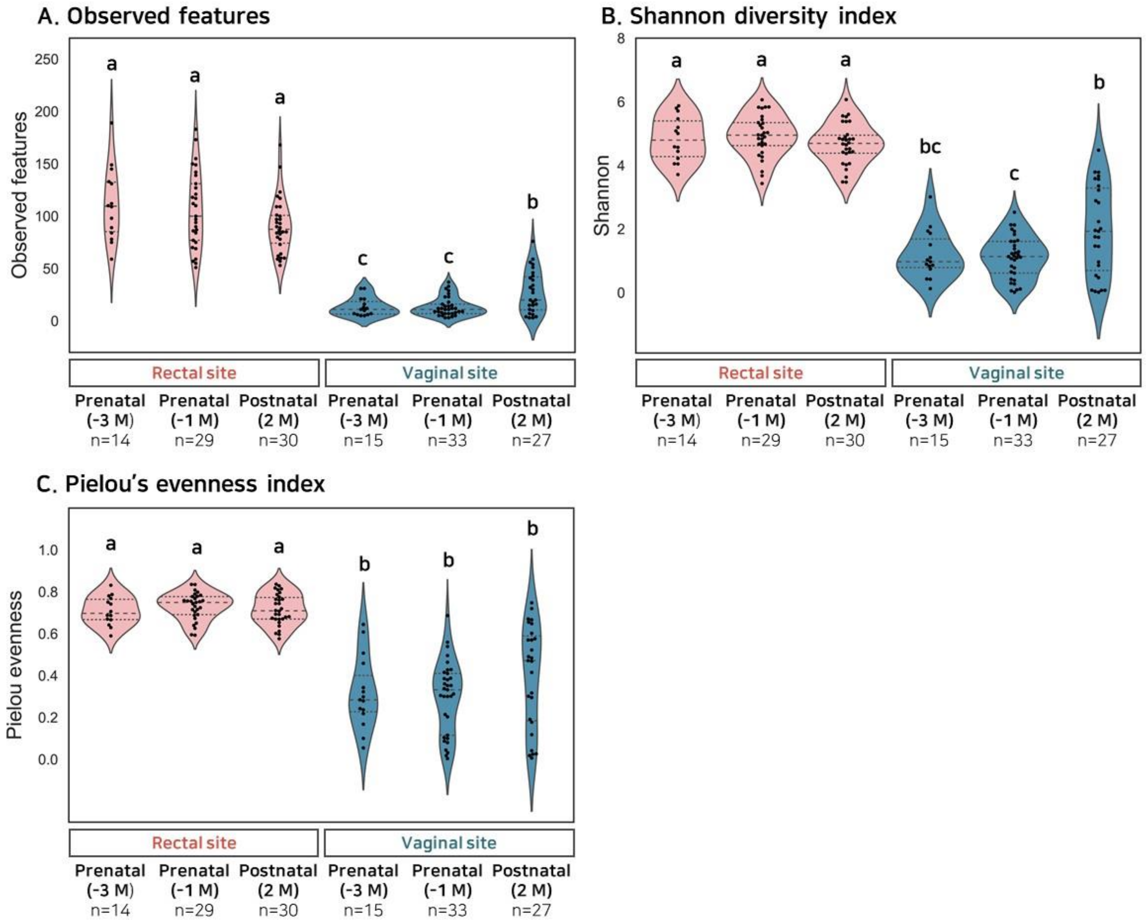

Supplementary Figure 1. Alpha diversity measures of pre- and post-partum rectal and vaginal microbiota. A) Observed features diversity, **B)** Shannon diversity, **C)** Pielou's evenness diversity. Labeled means without a common letter differ significantly, p-value < 0.05. Center line, median; quartile lines, upper and lower quartiles; violin plot, 1.5x interquartile range.

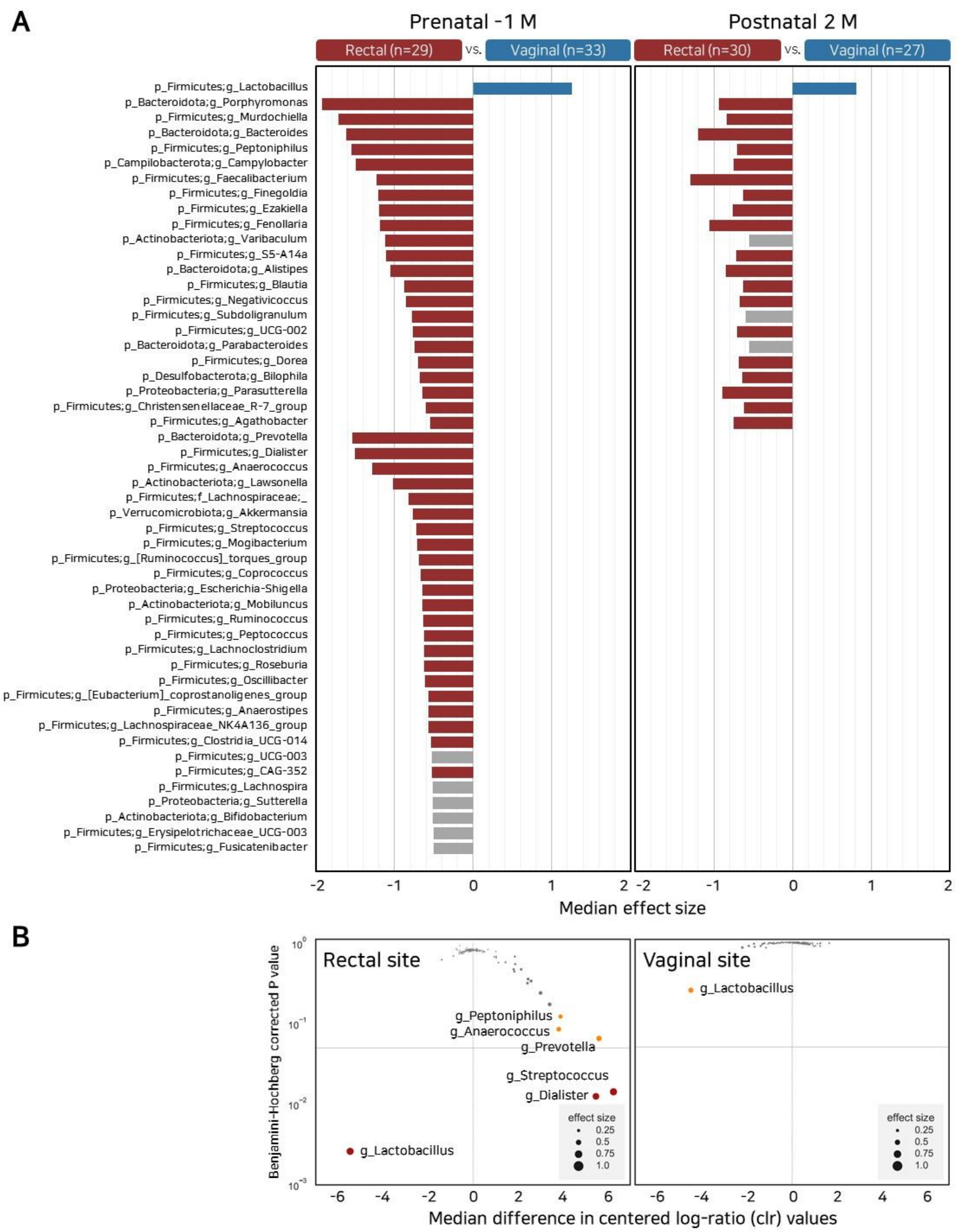

Supplementary Figure 2. Differences in the bacterial taxa of maternal pre- and post-partum rectal and vaginal sites determined by ALDEx2. Centered log-ratio transformed sequence read counts were used for statistical inference, and median effect size > 0.5 are shown. **A)** Differences of rectal versus vaginal sites. Features exceeding Benjamini-Hochberg corrected p-value (<0.05) thresholds are shown as red or blue, and features not exceeding p-value thresholds are shown as gray. **B)** Volcano plots showing differentially abundant features of prenatal versus post-partum bacterial taxa. Features are colored red if effect size (>0.5) and p-value (>0.05) thresholds are exceeded, and orange if only effect size (>0.5) threshold is exceeded.

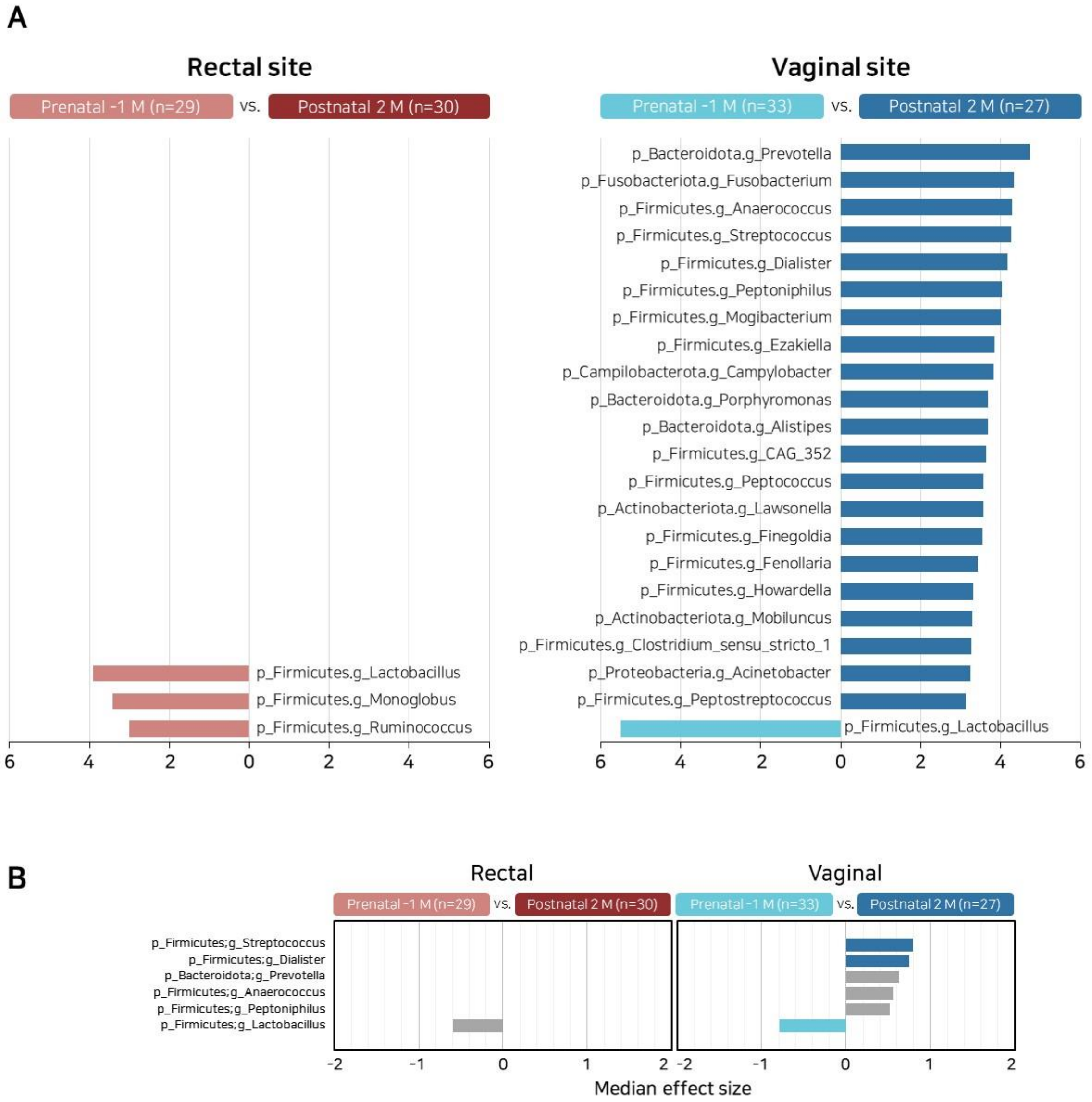

Supplementary Figure 3. Maternal rectal and vaginal sites; bacteria differing between pre-and post-partum determined by LEfSe (A) or ALDEx2 (B) analysis. A) LDA scores > 3.0 are shown. **B)** Features exceeding Benjamini-Hochberg corrected p-value (<0.05) thresholds are shown as red or pink, and features not exceeding p-value thresholds are shown as gray.

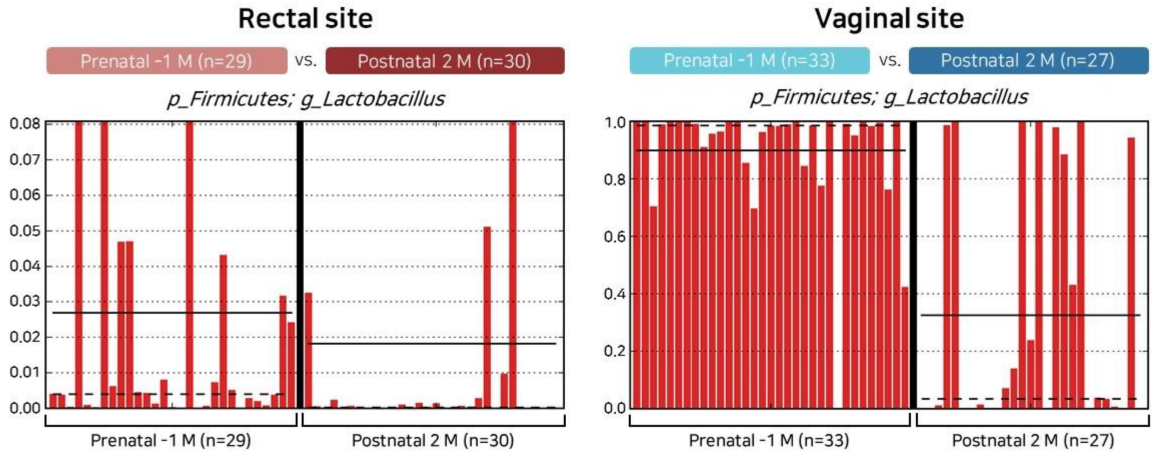

Supplementary Figure 4. Individual differences in *Lactobacillus* relative abundances by sampling time, measured by LEfSe analysis. Each column represents a single subject's *Lactobacillus* species.

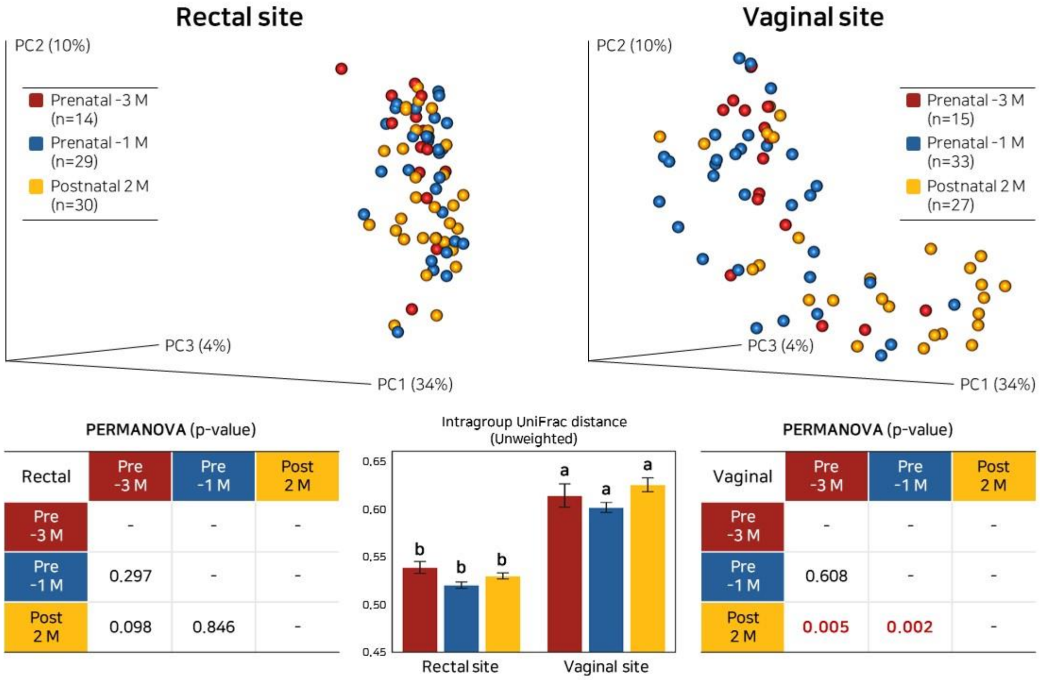

Supplementary Figure 5. Temporal changes in rectal and vaginal diversity during the last trimester and post-partum. PCoA plots using unweighted UniFrac distances. Labeled means without a common letter differ significantly, p-value < 0.001. PERMANOVA p-values listed in Punnet squares below each graph. Error bars represent mean ± SEM.

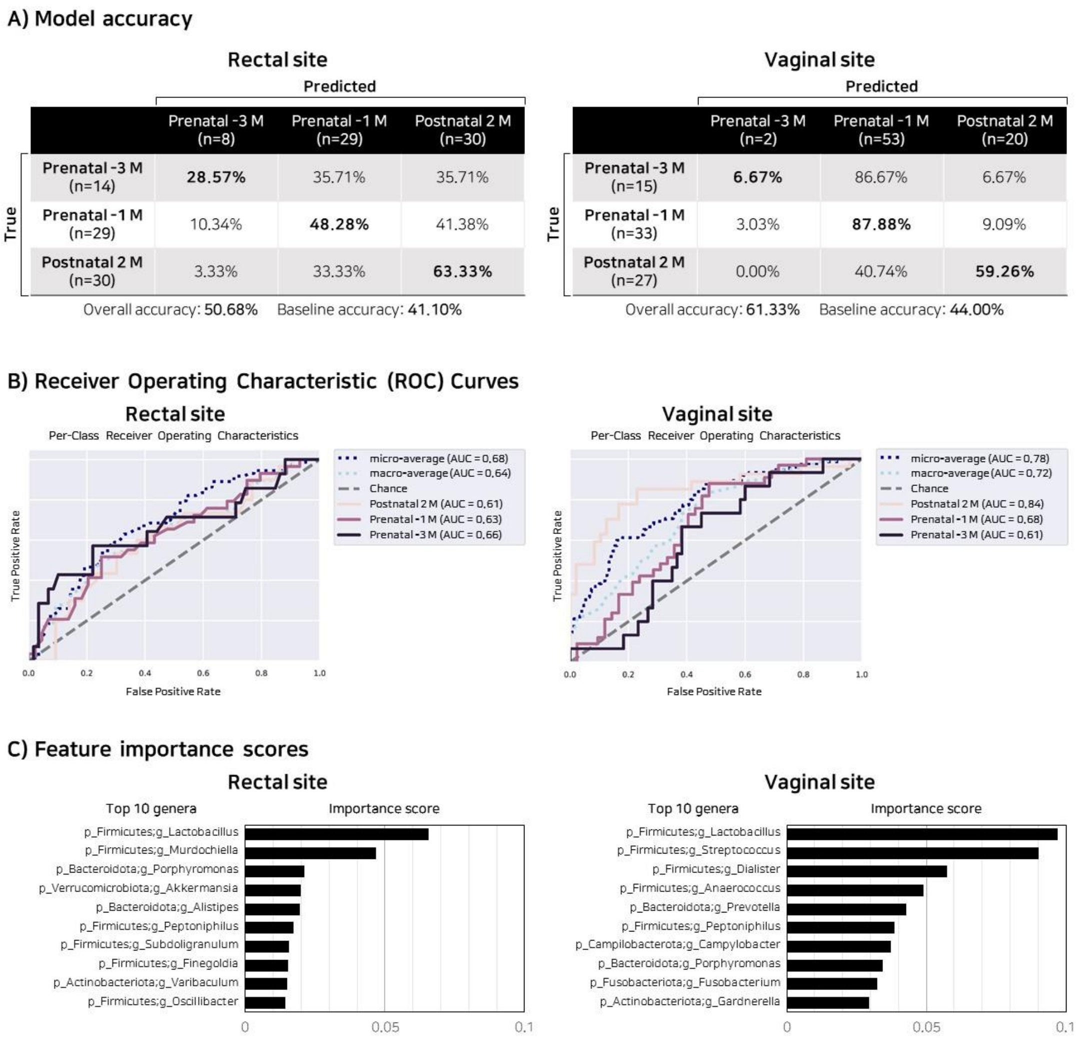

Supplementary Figure 6. Random Forest classifier of samples from maternal rectal and vaginal sites, between pre-and post-partum time-points based on the ASVs table, collapsed to genus level. A) The confusion matrix of the Random Forest classifier **B)** Receiver operation characteristics (ROC) analysis according to time-points. The area under the ROC curve (AUC) was calculated. **C)** The top 10 most predictive genera ordered by relative importance score used to assess the contribution of classifier accuracy.

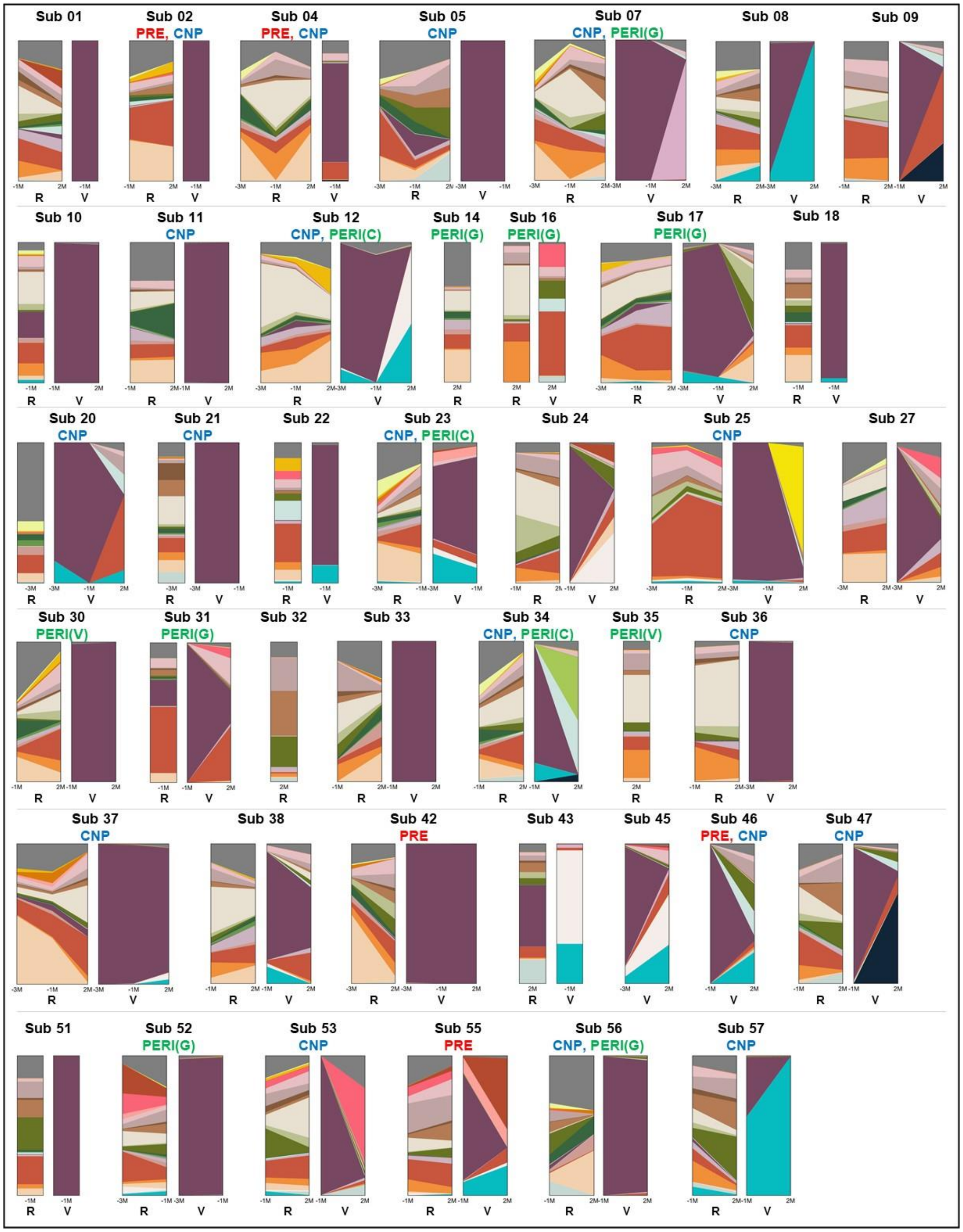

Supplementary Figure 7. Individual taxa plots of samples from maternal rectal (R) and vaginal (V) sites. PRE, Pre-natal antimicrobial exposure; CNP, C-section prophylaxis; PERI(G), Peri-natal antimicrobial exposure for Group-B Streptococcus prophylaxis; PERI(C), Peri-natal antimicrobial exposure for Chorioamnionitis, PERI(V), Peri-natal antimicrobial exposure for anti-viral treatment.
