# Supplementary material for: Partial convergence of the human vaginal and rectal maternal microbiota in late gestation and early post-partum
Source: NPJ Biofilms Microbiomes. 2023 Jun 13;9:37. doi: 10.1038/s41522-023-00404-5 (PMC10264455; doi:10.1038/s41522-023-00404-5)
Supplement: Supplementary file 2 — Reporting Summary [file 41522_2023_404_MOESM2_ESM.pdf]

nature portfolio

Corresponding author(s): Maria Gloria Dominguez Bello

Last updated by author(s): Apr 28, 2022

Reporting Summary

Nature Portfolio wishes to improve the reproducibility of the work that we publish. This form provides structure for consistency and transparency in reporting. For further information on Nature Portfolio policies, see our Editorial Policies and the Editorial Policy Checklist.

Statistics

For all statistical analyses, confirm that the following items are present in the figure legend, table legend, main text, or Methods section.

n/a	Confirmed	
☐	☒	The exact sample size (*n*) for each experimental group/condition, given as a discrete number and unit of measurement
☐	☒	A statement on whether measurements were taken from distinct samples or whether the same sample was measured repeatedly
☐	☒	The statistical test(s) used AND whether they are one- or two-sided<br>*Only common tests should be described solely by name; describe more complex techniques in the Methods section.*
☐	☒	A description of all covariates tested
☐	☒	A description of any assumptions or corrections, such as tests of normality and adjustment for multiple comparisons
☐	☒	A full description of the statistical parameters including central tendency (e.g. means) or other basic estimates (e.g. regression coefficient) AND variation (e.g. standard deviation) or associated estimates of uncertainty (e.g. confidence intervals)
☐	☒	For null hypothesis testing, the test statistic (e.g. *F*, *t*, *r*) with confidence intervals, effect sizes, degrees of freedom and *P* value noted<br>*Give P values as exact values whenever suitable.*
☐	☒	For Bayesian analysis, information on the choice of priors and Markov chain Monte Carlo settings
☒	☐	For hierarchical and complex designs, identification of the appropriate level for tests and full reporting of outcomes
☐	☒	Estimates of effect sizes (e.g. Cohen's *d*, Pearson's *r*), indicating how they were calculated

Our web collection on statistics for biologists contains articles on many of the points above.

Software and code

Policy information about availability of computer code

Data collection	No software/code was used .
Data analysis	QIIME 2 version 2020.6 was used for data analysis.

For manuscripts utilizing custom algorithms or software that are central to the research but not yet described in published literature, software must be made available to editors and reviewers. We strongly encourage code deposition in a community repository (e.g. GitHub). See the Nature Portfolio guidelines for submitting code & software for further information.

Data

Policy information about availability of data

All manuscripts must include a data availability statement. This statement should provide the following information, where applicable:

- Accession codes, unique identifiers, or web links for publicly available datasets
- A description of any restrictions on data availability
- For clinical datasets or third party data, please ensure that the statement adheres to our policy

The sequence data have been deposited in the European Nucleotide Archive under accession number ERP016173.

Field-specific reporting

Please select the one below that is the best fit for your research. If you are not sure, read the appropriate sections before making your selection.

☒ Life sciences ☐ Behavioural & social sciences ☐ Ecological, evolutionary & environmental sciences

For a reference copy of the document with all sections, see nature.com/documents/nr-reporting-summary-flat.pdf

Life sciences study design

All studies must disclose on these points even when the disclosure is negative.

Sample size	The study is a follow-up analysis of the previous longitudinal study (ECAM study, Bokulich et al. 2016) on aiming to determined the ante- and post-partum vaginal and rectal bacterial community structure in mothers. We sought to enroll at least 60 mothers, so that taking drop-outs into account, we would have a cohort of at least 40 babies who could be followed for at least one year. We assumed a Caesarian-section rate of ~50%, which would provide 20 babies in each group to examine the chief outcome variables. In total, 53 mothers were enrolled.
Data exclusions	Inclusion criteria included healthy pregnant mothers aged 18-45. For better generalizability of the results and to reduce the likelihood of loss to follow-up, we excluded mothers who were at high risk for premature delivery (i.e. incompetent cervix, premature labor, bed rest), had fetal anomalies detected in utero, and/or were unable to meet study obligations. We excluded infants who required treatment in a neonatal intensive care unit greater than 7 days, premature infants of less than 38 weeks gestation, and those with genetic diseases an/or birth-related injuries requiring intensive monitoring.
Replication	All microbiota analysis were repeated with different sampling depth values (4,000-20,000).
Randomization	Randomization was not relevant to this study, since the aim of this study is to examine the development of the intestinal microbiota in early childhood.
Blinding	Blinding was not relevant to this study.

Reporting for specific materials, systems and methods

We require information from authors about some types of materials, experimental systems and methods used in many studies. Here, indicate whether each material, system or method listed is relevant to your study. If you are not sure if a list item applies to your research, read the appropriate section before selecting a response.

Materials & experimental systems

n/a	Involved in the study	
☒	☐	Antibodies
☒	☐	Eukaryotic cell lines
☒	☐	Palaeontology and archaeology
☒	☐	Animals and other organisms
☐	☒	Human research participants
☒	☐	Clinical data
☒	☐	Dual use research of concern

Methods

n/a	Involved in the study	
☒	☐	ChIP-seq
☒	☐	Flow cytometry
☒	☐	MRI-based neuroimaging

Human research participants

Policy information about studies involving human research participants

Population characteristics	Inclusion criteria included healthy pregnant mothers aged 18-45. For better generalizability of the results and to reduce the likelihood of loss to follow-up, we excluded mothers who were at high risk for premature delivery (i.e. incompetent cervix, premature labor, bed rest), had fetal anomalies detected in utero, and/or were unable to meet study obligations. We excluded infants who required treatment in a neonatal intensive care unit greater than 7 days, premature infants of less than 38 weeks gestation, and those with genetic diseases an/or birth-related injuries requiring intensive monitoring.
Recruitment	Enrollment of mothers, which was specific for this study, began in December 2011 and ended in December of 2014 at New York University School of Medicine. Participation was voluntary and included written informed consent.
Ethics oversight	This study was approved by the Institutional Review Board at the NYU School of Medicine.

Note that full information on the approval of the study protocol must also be provided in the manuscript.
